# Construction Project Safety Performance Management Using Analytic Network Process (ANP) as a Multicriteria Decision-Making (MCDM) Tool

**DOI:** 10.1155/2020/2610306

**Published:** 2020-02-25

**Authors:** Murat Gunduz, Basil K. Khader

**Affiliations:** Dept. of Civil Engineering, Qatar University, Po Box: 2713, Doha, Qatar

## Abstract

The paper addresses the context in which the construction industry is considered risky, as the intense labor and machine environment interacts with acceleration and overlapping activities. This situation results in accidents and fatalities. A high number of accidents and fatalities leads to additional costs and delays, detrimental to all stakeholders. Hazard identification and quantification of their impacts on building safety are crucial for planning. Classifying security risks is a complex process, and risks are interconnected. There is a gap in the literature to study the interconnections of these hazards along with the frequency of occurrences. To bridge this gap, the frequency-adjusted importance index and the ANP (Analytical Networking Process) tool were used to capture the 14 interconnections and their frequencies based on the results of a survey distributed to 106 construction professionals. The main contribution of this work to existing knowledge is to identify and prioritize potential risks in the construction sector, considering their interconnections and their level of occurrence frequency. This is the first study in the literature to combine the frequency-adjusted importance index and the ANP tool, both integrated. The results from the importance index was used as the base for pairwise comparison for the ANP model. Based on the results from the model, recommendations to industry professionals are provided and presented.

## 1. Introduction

Construction industry is one of the biggest industries worldwide. Deplorable safety management practices are damaging the reputation of the construction sector [1]. Due to the increasing complexity of construction projects, the construction industry is acknowledged as having inherent risks with high levels of change and uncertainty [2]. Many workers and equipment interact together to deliver the final project on time. This interaction would mostly result in accidents. Safety performance monitoring should be performed by all stakeholders to avoid injuries and fatalities in the construction sites.

The first step in evaluating the safety performance of a construction site is to identify the hazards, evaluate their priorities and effect, and take adequate measures to avoid such hazards. Due to the dangers of the construction industry, leading and lagging safety indicators have been developed to measure safety performance and prevent injury [3]. The application of BIM is currently experiencing rapid growth in construction operations and planning and management, as well as in safety management [4]. New technologies are also used to identify hazards such as BIM (Building Information Modeling) [5]. However, evaluating the priorities of the hazards is a complex issue as many of these hazards are interrelated to each other. Traditional risk analysis methods are not efficient to analyze nonlinear or complex systems such as construction sites [6]. There is a gap in the literature to study the interconnections of these hazards along with their frequency of occurrences. To cover this gap, frequency-adjusted importance index and ANP (Analytic Network Process) tool were jointly utilized to capture the interconnections and their frequencies based on the results from a survey distributed to construction professionals. The main contribution of this paper to the existing knowledge is to identify and prioritize potential hazards in the construction sector by considering their interconnections along with their frequency level of occurrences. Although very few researchers used ANP to study construction safety hazards [7–9], they failed to address the frequency component. This is the first study in the literature to introduce frequency component to the ANP tool for realistic capturing of hazard rankings.

After literature review, 42 hazards in 14 categories were identified. These were presented in a survey and distributed online to the construction industry experts. 106 responses were received, analyzed, and ranked using frequency-adjusted importance index and ANP. The results from frequency-adjusted importance index were used as the base for pairwise comparison for the ANP model. The ANP tool reflected the interdependencies between the safety hazards. With the help of ANP, the hazards are linked together in an analytic network to reach a robust model and outcome. The ranked hazards are presented and proper recommendations were made to industry professionals based on the research outputs.

## 2. Literature Review

There is a growing body of the literature on assessing perceptions of safety climate [10]. Construction sites are known for its complex environments where many unsafe acts and/or unsafe conditions exist [6].

Identifying hazards was performed in many studies such as [11–18]. Gunduz et al. [19] conducted an extensive literature review to identify 168 observable variables in 16 latent dimensions that affect safety. The study then proposed a multidimensional safety performance model utilizing structural equation modeling (SEM) analysis. Analytic hierarchy process (AHP) then was used to assess the severity of each. On the other hand, Esmaeili et al.[20] adopted a preconstruction safety management in which they have identified 22 fundamental attributes with highest impact on safety to predict the safety outcome.

Traditional risk analysis methods are not efficient to analyze nonlinear or complex systems such as construction sites [6]. Traditional risk analysis methods are not efficient to analyze nonlinear or complex systems such as construction sites [6].

Few studies in the literature adopted the use of ANP to rank hazards. Yang et al. [8] assessed metro construction safety risk by the use of ANP-grey clustering method. Zhou et al. [9] assessed high-risk hydropower-construction project work system hazards by the analytic network process (ANP) and decision-making trial and evaluation laboratory (DEMATEL). High-rise construction safety culture among job positions with Fuzzy ANP and Fuzzy Decision Trail and Evaluation Laboratory and methods was studied by [7]. The past studies usually study one type of construction without considering their frequency of occurrence on the construction site. There is a gap in the literature to study the interconnections of safety hazards along with the consideration of their frequency of occurrences. This study covers this gap in the literature by considering ANP modeling and the frequency of safety hazards together. With the help of ANP, the hazards are linked together in an analytic network to reach a robust model and outcome. In this study, an extensive literature review on topics related to safety hazards in construction projects was conducted. A draft checklist of 42 hazards in 14 categories (site planning and housekeeping; management involvement; handling, storage, and use of materials; welding and cutting; concrete and concrete framework; crane and lifting equipment; electrical equipment; hand and power tools and machinery; working at height and protection against falling; personal protective equipment; traffic and transportation control; scaffolding and ladders; fire prevention; excavation, trenching, and shoring) were collected.  Table 1 presents the top forty-two hazards utilized in the study with their corresponding references. Based on the identified safety hazards, a questionnaire was prepared and distributed among the construction professionals. The questionnaire helped identify the most significant safety hazards in the construction industry.

Next sections will introduce the methodology on frequency-adjusted importance index, ANP, and the data analysis parts.

## 3. Methodology

**Figure 1:**
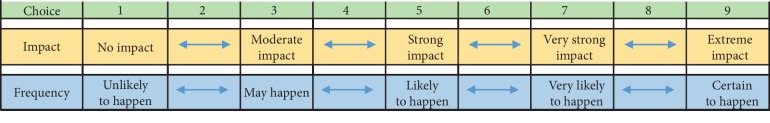
Scale definition for the importance and frequency levels for each hazard.

This study gathered a list of 42 safety hazards from the literature review. A survey approach was adopted as means of collecting data for the data analysis. The survey aimed to investigate perceptions of the respondents on safety hazards attributes in the construction industry. A ranking analysis was applied between respondents based on their organization type, job designation, industry type, total construction experience, and size of their companies. The respondents were requested to evaluate the attributes based on a (1–9) scale for the importance and frequency levels of the factors. The (1–9) scale is depicted in Figure 1. The survey was sent to construction professionals that play key roles in the construction industry worldwide.

A total number of 106 completed surveys was received from the respondents worldwide. Frequency-adjusted importance index and ANP analysis were applied on the collected data. Ranked attributes were interpreted based on the statistical analyses performed. Finally, recommendations to industry professionals were carried based on the outcomes of the data analysis.

## 4. Data Characteristics

An online website tool (SurveyMonkey) was employed in developing, distributing the questionnaire, and collecting responses. Moreover, hardcopies were also distributed to authors' networks. The motivation of the respondents was to receive the outcome of this study after its completion. The main results came from international construction companied based in Qatar, the USA, Japan, Turkey, and Jordan. Respondents are from different companies with different functions, departments, and projects/project types. They mainly are construction engineers, managers, safety supervisions, design engineers, consultants, and owners. The questionnaire consists of 20 questions. The first 6 are related to the respondents' location, organization type and size, construction type, job designation, and total years of experience. Other 14 questions are related to scaling (1 to 9) of each hazard in terms of impact and frequency.

The questionnaire link was sent out by emails or via professional networks worldwide. Data collection resulted in 106 completed questionnaires. Contractors are the largest portion of respondents with 58 responses (54.7%). Consultants, the second largest contributors of the survey, form almost 17% of the total participants. Project engineer and project/construction managers make a total of 60% of the responses. Participants involved in infrastructure and oil and gas construction projects hold the significant portion of participants with 28% and 23% of responses, respectively. Participants were categorized based on total years of work experiences in construction based on four groups, which are 0 to 5 years, 6 to 10, 11 to 15, and more than 16 years. A percentage of 40% of responses was yielded from professionals with experience between 6 and 10 years.

## 5. Data Analysis

One of the objectives of this paper is to get the perceptions of the construction professionals about the major safety hazards in construction projects. Survey participants rated each hazard importance and frequency based on a (1–9) scale. Participants were asked to evaluate the importance (the impact) of the hazard on safety performance. Frequency was also rated in order to decide on how often the hazard is come across in construction projects.

As an example, considering the factor “use of weak and deformed forms,” the questions to the respondent related to this hazard are

- What is the impact of “use of weak and deformed forms” on safety performance?
- How often the “use of weak and deformed forms” would likely to happen on a construction site?

The survey was sent to construction industry professionals. The data analysis is presented in the following sections.

### 5.1. Frequency-Adjusted Importance Index (FAII)

A similar yet inventive ranking approach adopted in this research to rank safety attributes in the construction industry is the Frequency-Adjusted Importance Index (FAII) [35]. This technique considers both the importance and the frequency. In order to calculate the FAII, both the relative importance index (RII) and the frequency index (FI) are required. The equations for FAII, RII, and FI are shown below:

(1) $$\begin{array}{c} \text{RII}\left(\%\right)=\frac{{\sum I}^{}}{A\left(N\right)}\times 100,\\ \text{FI}\left(\%\right)=\frac{{\sum F}^{}}{A\left(N\right)}\times 100, \end{array}$$

where *I* = weight given to importance by the respondent (1 to 9), *F* = weight given to frequency by the respondents (1 to 9), *A* = the highest weight (in this case 9), and *N* = total number of respondents (in this case 106)

Based on both the RII (%) and FI% equations, the frequency-adjusted importance index will be calculated as follows:

(2) $$\begin{array}{c} \text{FAII}\left(\%\right)=\frac{\left(\text{RII}\left(\%\right)\times \text{FI}\%\right)}{100}. \end{array}$$

FAII provides better ranking results because it reflects the effects of importance and frequency all together.

### 5.2. Analytic Network Process (ANP)

The ANP came as a generalized form of the AHP as many decision problems cannot be dealt with as a linear hierarchy structure. This is because of the existence of interdependences and interaction between the factors. While AHP depends on a hierarchical form of levels of goal, criteria, and subcriteria, the ANP deals with all factors as clusters in a network, which are all connected to the main goal (safety performance in this paper). The other advantage of the ANP is the network connecting the clusters and its elements together.

The safety performance in construction is a complex decision problem as many factors are interrelated. Hazards of a certain nature and under a certain category can have obvious influence and can develop other risks in a different category. Hence, it cannot be dealt as independent criteria. Similarly, hazards in a certain category can influence hazards under the same category. This is called the inner dependence, while the previously described relation between categories is called the outer dependence. In such problems, ANP would be a very powerful alternative to AHP and other methods.

**Figure 2:**
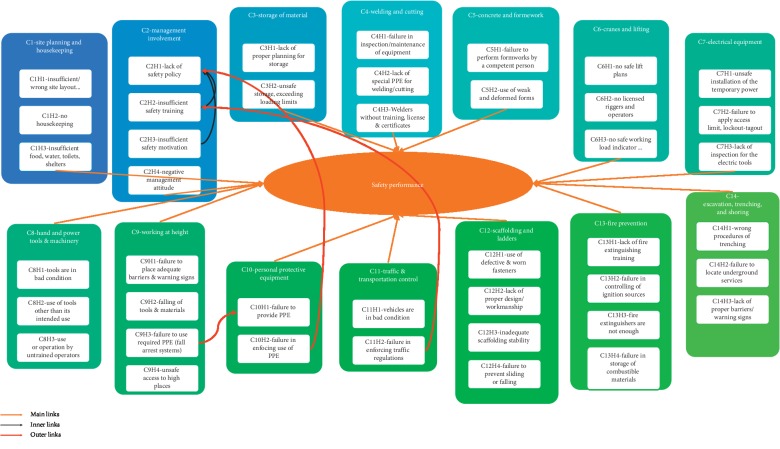
ANP safety performance model with relevant categories.

Figure 2 illustrates the proposed ANP model. The model is a network that consists of categories called clusters. Each cluster contains the elements, which are called the hazards. The safety performance is connected to all clusters. Until this stage, the model is linear and categories are independent. The red and black arrows represent the interdependences, which is the nonlinear part of the model. As explained, these show the effect of relevant hazards in influencing others. As an example, the C2H1, “lack of safety policy” has its influence on C10H2, “failure in enforcing use of PPE.”

**Figure 3:**
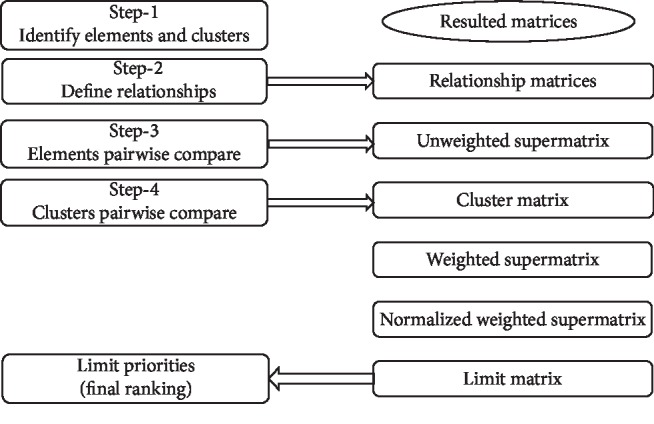
ANP implementation steps and resulting matrices at each step.

**Figure 4:**
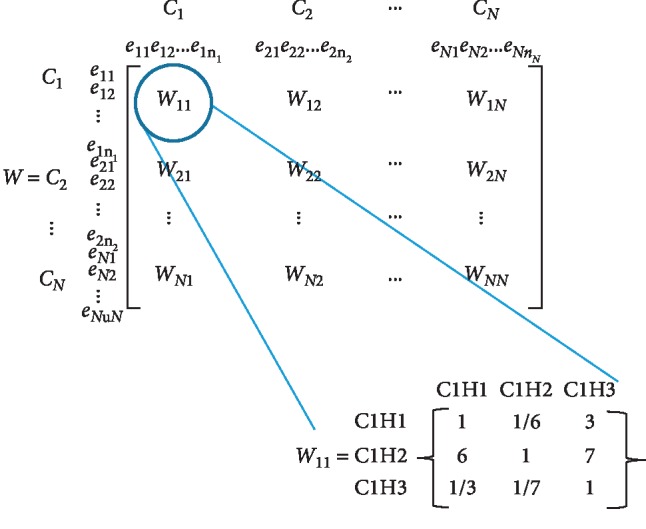
The supermatrix formulation.

The steps to implement ANP model can be seen in Figure 3. Steps one and two are presented in Figure 2. The 3rd step is to develop a pairwise comparison between the elements in a matrix format and then to put these submatrices together to form the unweighted supermatrix (Figure 4). The pairwise comparison is calculated based on FAII ranking of factors. As an example, the FAII rank of C1H2 is 5, while the FAII rank of C1H3 is 29. The difference in ranking is 24. Using linear interpolation to scale the differences in a (1–9) scale by considering the maximum rank difference of 31 (maximum rank difference is between factors C4H1 and C4H2), the result will be 7/9. The scaling table can be seen in Table 2. And this is inserted in the W11 matrix in Figure 4. The rest of the pairwise comparison was similarly carried out.

The fourth step is to do pairwise comparison at the cluster level to develop the cluster matrix. The weight of the cluster is determined by the weights of its components, which are the nodes (in this case “hazards”). The average value of the hazard weight in FAII was already calculated for the main category. As an example, weight of C1 = (C1H1 + C1H2 + C1H3)/3, which is (0.32404 + 0.35800+0.29878)/3 = 0.3269. Similarly, cluster weights were calculated for all main categories.

Multiplication of each block in the unweighted supermatrix by the weight of the corresponding cluster weight will result in the weighted supermatrix. Raising the weighted supermatrix to high power will make it convergent as the limiting matrix. The results are the rank of the hazards, which is given by the priority vector in the limit matrix. These calculations can be carried out with the help of software such as SuperDecision. The result of FAII and ANP can be seen in Table 3 below.

From Table 3, it was seen that the top 5 ranked safety hazards based on ANP results are (1) lack of company's safety policy, (2) insufficient safety training, (3) failure in enforcing, motivating, and training workers to use PPE, (4) no housekeeping (scattered garbage and material, dusts, excessive noise, vibration, etc.), and (5) insufficient safety motivation and incentives.

### 5.3. Safety Performance Index (SPI)

The previous results will be utilized to measure the safety performance in construction sites. The 42 hazards will be used to measure safety performance in construction sites. These hazards will be used for calculating SPI (safety performance index), which then can be used to measure safety performance in construction sites, compare sites together, and benchmarking.

The main idea is to do site inspection focusing on these hazards and evaluate if the site under inspection is complying with the safety procedures to avoid such hazard. Compliance will be given a weight of 100% and noncompliance will be 0%. Then, the safety index will be the site compliance for each hazard, multiplied by the limiting vector of the hazards.

The SPI can be given according to the following formula:

(3) $$\begin{array}{c} \text{SPI}=\Sigma L.E\text{,} \end{array}$$

where *L* is the limiting vector resulted from the ANP, which is normalized for all hazards, and the summation will equal to 1, and *E* is site evaluation of each hazard (0–100%) measured by a safety expert.

However, the formula is not considering that some of the hazards can be not applicable in some construction sites due to the type of construction. As an example, welding is not considered as hazard in the building site and concrete work can be ignored in a mechanical pipeline project. In such cases, inapplicable hazards are ignored, and then all other hazards will be normalized to the new summation.

The new limiting vector can be called Ln (normalized limiting vector). The final general formula will be

(4) $$\begin{array}{c} \text{SPI}=\Sigma L_{n}.E\text{,} \end{array}$$

where *L*_*n*_ is the normalized limiting vector and E is site evaluation of each hazard (0–100%) measured by safety expert.

As an example, refer to Table 4 below. It shows the calculation of an SPI for a random construction site. The safety index is found to be 83.7%. The same table is showing the safety index of each main category. C1-“site planning and housekeeping” is 58.3 and C2-“management involvement” is 88.4, etc. that some hazards are not applicable. Each category's SPI index was calculated by the formula below. This calculation helps the construction team to take action against each category:

(5) $$\begin{array}{c} \text{SPI}\left(\text{each category}\right)=\frac{\Sigma L_{n}.E}{\Sigma 100*L_{n}}. \end{array}$$

## 6. Discussion of Results and Practical Implications

42 hazards in 14 categories were identified and offered in a survey after reviewing the literature. The survey was distributed to construction industry professionals. 106 respondents assessed the 42 hazards based on impact (the hazard impact on safety performance in construction projects) and frequency (how often the hazard is likely to happen). The collected data of 106 responses were then analyzed by frequency-adjusted importance index.

The resulted ranking of the hazards was then utilized to perform ANP (Analytic Network Process) as a second stage ranking tool in a purpose to reveal the root causes of these hazards. The ANP was selected as it is a powerful multicriteria decision-making technique for complex problems. The complexity is due to the existence of interdependencies between hazards from or across different categories.

It can be concluded from Table 3 that the hazards, which is considered most significant, is the “lack of company's safety policy” (ANP rank 1). This is related to the organization safety management at the planning phase of the project. The safety policy is a strong evidence of commitment toward safety and the methods to implement safety procedures on-site. It is to be noted that this ranked 19^th^ in FAII. This result shows the strength of the ANP technique in representing the real causes, or the latent hazards, which stand behind many hazards. “Insufficient safety training” (ANP rank 2) is another hazard under the management category. This hazard ranked the first in FAII, and to which most of the accident in construction is referred. This hazard is explicit and latent, as many other hazards are connected to it. “Failure in enforcing, motivating, and training workers to use PPE” ranked as third in ANP compared to seventh in FAII. This is also considered as a latent or causing hazard of many hazards related to using PPE such as the “failure to use required PPE (fall arrest systems) and safety nets,” which ranked 4th in FAII.

In this study, it has been proved that safety experts shall focus their attention to the root cause of the hazards, that is, the latent hazards, which actually drive the accidents and injuries. However, focusing on solving the apparent hazards in a reactive way would not improve safety performance and will keep such hazards repeating as long as construction is ongoing. Hence, the most important is to solve the root causes of the problems.

From the study, it has been found that management involvement is the most important factor in improving the safety performance by adopting a robust clear safety policy, which shall include safety and craft training, motivation and incentives, and enforcing and accountability toward safety in all levels of the work force. This proactive attitude will help make safety as a culture at the construction sites. Therefore, the recommendation to construction industry leadership is to focus on safety policies and management commitment to safety when selecting their stakeholders of consultants and contractors.

Furthermore, this paper recommends safety experts to identify hazards, prioritize them, and distribute the budget wisely to prevent accidents.

## 7. Conclusion

The construction industry is considered risky as labor and machinery intense environment interacts with accelerating and overlapping activities. This situation would result in high number of accidents and fatalities. High number of accidents and fatalities lead to additional cost and delay on all stakeholders including public agencies, project owners, development companies, consultants, and construction companies. Identifying hazards and quantifying their impacts on construction safety are crucial for planning, budgeting, and management purposes. Safety hazards ranking is a complex process as these hazards are interconnected. There is a gap in the literature to study the interconnections of these hazards along with their frequency of occurrences. This is the first study in the literature to combine frequency adjusted importance index and ANP tool together. Past literature conducted targeting the safety performance evaluation were focusing on identifying the observable hazards and evaluating their apparent effects. A frequency-adjusted importance index analysis was carried out in this paper as a first stage by ranking the hazards. The top three hazards according to FAII were (1) insufficient safety training, (2) negative management attitude to safety, and (3) insufficient safety motivation and incentives.

A second stage ranking was carried out by using the ANP (Analytic Network Process). This technique has proven its benefits in solving complex decision problems due to existence of interdependences between its parts, which is the case in safety hazards where some hazards are interrelated. The ANP ranking gave a close ranking similar to FAII where the top three hazards were (1) lack of company's safety policy, (2) insufficient safety training, and (3) failure in enforcing, motivating, and training workers to use PPE.

The results of both analyses confirm that the role the management plays is an important role to improve the safety performance by establishing a safety policy, adopting safety-training procedures, enforce safety procedures through incentives, and control measures.

The outcome of this paper would help the construction and the safety professionals on assessing and quantifying the most critical safety hazards in the construction industry. Moreover, the construction and safety professionals would utilize the safety performance index calculation to quantitatively measure their site safety level.

This study could be extended further by developing a practical tool to measure the safety performance index and conducting case studies on comparison of the safety performance index in construction projects.

## Figures and Tables

**Table 1:** The top 42 hazards from the literature review and their relevant references.

Code	Description	References
C1	Site planning and housekeeping	
C1H1	Insufficient working spaces/wrong site layout/no access/no lights	[20–26]
C1H2	No housekeeping (scattered garbage and material, dusts, excessive noise, vibration, etc.)	
C1H3	Insufficient food, drinking water, toilets, rest shelters, and medical facilities	

C2	Management involvement	
C2H1	Lack of company's safety policy	[12, 20, 24, 25, 27–31]
C2H2	Insufficient safety training	
C2H3	Insufficient safety motivation and incentives	
C2H4	Negative management attitude to safety	

C3	Handling, storage, and use of materials.	
C3H1	Lack of proper planning and workforce for storage	[32–34]
C3H2	Unsafe storage/stacking of materials and exceeding safe loading limits	

C4	Welding and cutting	
C4H1	Failure in handling, inspection, and maintenance of equipment (cylinders, machines, hoses, and cables)	[19, 34–37]
C4H2	Lack of special PPE (personal protective equipment), such as face shield, special gloves, and goggles for welding/cutting	
C4H3	Welders without training, license, and certificates	

C5	Concrete and concrete framework	
C5H1	Failure to perform form works under the supervision of a competent person	[33, 38]
C5H2	Use of weak and deformed forms	

C6	Crane and lifting equipment	
C6H1	Unavailability of a safe lift plan on-site	[34, 37–40]
C6H2	Lack of licensed trained riggers and operators	
C6H3	Lack of safe working load indicator/inspection stickers/latches/barricades	

C7	Electrical equipment	
C7H1	Unsafe installation of the temporary power (old, damaged, and wrong rating of panels, sockets, wires, etc.)	[19, 33, 34, 37]
C7H2	Failure to apply access limit, lockout-tagout, permit system, and signage systems	
C7H3	Lack of inspection for the electric tools, cables, and equipment	

C8	Hand and power tools and machinery	
C8H1	Tools are in bad condition with no regular inspection	[20, 24, 33, 34, 41]
C8H2	Use of tools other than its intended use	
C8H3	Use or operation by untrained and unauthorized operators (lack of training system)	

C9	Working at height and protection against falling	
C9H1	Failure to place adequate barriers and warning signs for open edges and holes	[19, 21, 22, 34, 38]
C9H2	Falling of hand tools and other materials	
C9H3	Failure to use required PPE (fall arrest systems) and safety nets	
C9H4	Unsafe access to high places by damaged ladders, lifts, etc.	

C10	Personal protective equipment	
C10H1	Failure to provide appropriate and adequate personal protective equipment for workers (head, eye, face, hand, foot, and hearing protection)	[31, 41, 42]
C10H2	Failure in enforcing, motivating, and training workers to use them.	

C11	Traffic and transportation control	
C11H1	Vehicles (buses/pickups/trucks/others) are in bad condition and do not have regular maintenance/first aid equipment/fire extinguishers/lights	[20, 34, 38, 43]
C11H2	Failure in enforcing traffic regulations (seat belt, speed limit, license, and training)	

C12	Scaffolding and ladders	
C12H1	Use of defective and worn fasteners, components, settings, and material in scaffolding system	[37, 38, 44, 45]
C12H2	Lack of proper design, workmanship, and regular inspections	
C12H3	Inadequate scaffolding stability (guardrails, toe boards, secured ties, etc.)	
C12H4	Failure to provide safe access against slipping, sliding, or falling	

C13	Fire prevention	
C13H1	Lack of fire extinguishing training, escape plans, and drills	[19, 34, 37, 46]
C13H2	Failure in controlling of ignition sources and fire watches, fire blankets, etc	
C13H3	Fire extinguishers are not enough, not in proper locations, not accessible, and not regularly maintained	
C13H4	Failure in storage of flammable liquids and combustible materials	

C14	Excavation, trenching, and shoring	
C14H1	Wrong procedures (with slab, timber, trench, boxes, shoring, lining, etc.)	[19, 38]
C14H2	Failure to locate underground services and to take precautionary measures	
C14H3	Lack of proper barriers/warning signs/lights for the excavation	

**Table 2:** Scaling table through linear interpolation based on the differences between the factors.

Difference	(1–9) scale	Difference	(1–9) scale
1	2	17	6
2	2	18	6
3	2	19	6
4	3	20	6
5	3	21	7
6	3	22	7
7	3	23	7
8	4	24	7
9	4	25	8
10	4	26	8
11	4	27	8
12	5	28	8
13	5	29	9
14	5	30	9
15	5	31	9
16	5	32	9

**Table 3:** FAII (%) values and ranking of safety attributes by ANP.

CODE	Name	FAII	Rank 1	ANP	Rank 2↓
C2H1	Lack of company's safety policy	0.28753	19	0.271236	1
C2H2	Insufficient safety training	0.40116	1	0.080684	2
C10H2	Failure in enforcing, motivating, and training workers to use PPE	0.32259	7	0.074344	3
C1H2	No housekeeping (scattered garbage and material, dusts, excessive noise, vibration, etc.)	0.33981	5	0.054578	4
C2H3	Insufficient safety motivation and incentives	0.39506	2	0.052904	5
C9H3	Failure to use required PPE (fall arrest systems) and safety nets	0.34568	4	0.051955	6
C2H4	Negative management attitude to safety	0.38752	3	0.03552	7
C9H2	Falling of hand tools and other materials	0.32693	6	0.032775	8
C14H2	Failure to locate underground services and to take precautionary measures	0.31173	11	0.031273	9
C11H2	Failure in enforcing traffic regulations (seat belt, speed limit, license, and training)	0.30476	13	0.0285	10
C13H4	Failure in storage of flammable liquids and combustible materials	0.31005	12	0.027115	11
C6H3	Lack of safe working load indicator/inspection stickers/latches/barricades	0.31981	8	0.02209	12
C9H1	Failure to place adequate barriers and warning signs for open edges and holes	0.31855	9	0.019644	13
C4H2	Lack of special PPE (personal protective equipment), such as face shield, special gloves, and goggles for welding/cutting	0.31741	10	0.019333	14
C7H1	Unsafe installation of the temporary power (old, damaged, and wrong rating of panels, sockets, wires, etc.)	0.2758	24	0.018633	15
C8H3	Use or operation by untrained and unauthorized operators (lack of training system)	0.29729	15	0.01509	16
C10H1	Failure to provide appropriate and adequate personal protective equipment for workers (head, eye, face, hand, foot, and hearing protection)	0.28235	20	0.014869	17
C13H3	Fire extinguishers are not enough, not in proper locations, not accessible, and not regularly maintained	0.29127	17	0.014117	18
C14H3	Lack of proper barriers/warning signs/lights for the excavation	0.28929	18	0.01344	19
C3H2	Unsafe storage/stacking of materials and exceeding safe loading limits	0.29321	16	0.012805	20
C1H1	Insufficient working spaces/wrong site layout/no access/no lights	0.28045	22	0.012462	21
C7H2	Failure to apply access limit, lockout tag-out, permit system, and signage systems	0.26612	26	0.011738	22
C12H3	Inadequate scaffolding stability (guardrails, toe boards, secured ties, etc.)	0.2809	21	0.009772	23
C9H4	Unsafe access to high places by damaged ladders, lifts, etc.	0.30041	14	0.008982	24
C7H3	Lack of inspection for the electric tools, cables, and equipment	0.26452	27	0.007395	25
C6H2	Lack of licensed trained riggers and operators	0.27984	23	0.005696	26
C1H3	Insufficient food, drinking water, toilets, rest shelters, and medical facilities	0.26155	29	0.005691	27
C13H2	Failure in controlling of ignition sources and fire watches, fire blankets, etc.	0.26296	28	0.005473	28
C5H1	Failure to perform form works under the supervision of a competent person	0.21498	39	0.005426	29
C12H4	Failure to provide safe access against slipping, sliding, or falling	0.27511	25	0.005256	30
C11H1	Vehicles (buses/pickups/trucks/others) are in bad condition and do not have regular maintenance/first aid equipment/fire extinguishers/lights	0.25774	31	0.00475	31
C4H3	Welders without training, license, and certificates	0.2516	34	0.003663	32
C14H1	Wrong procedures (with slab, timber, trench, boxes, shoring, lining, etc.)	0.25274	33	0.003465	33
C8H2	Use of tools other than its intended use	0.24863	35	0.00301	34
C13H1	Lack of fire extinguishing training, escape plans, and drills	0.25591	32	0.002861	35
C5H2	Use of weak and deformed forms	0.19334	42	0.002713	36
C6H1	Unavailability of a safe lift plan on-site	0.25873	30	0.002448	37
C3H1	Lack of proper planning and workforce for storage	0.20988	40	0.001829	38
C12H2	Lack of proper design, workmanship, and regular inspections	0.23285	36	0.001809	39
C8H1	Tools are in bad condition with no regular inspection	0.21674	38	0.001801	40
C4H1	Failure in handling, inspection, and maintenance of equipment (cylinders, machines, hoses, and cables)	0.19444	41	0.00162	41
C12H1	Use of defective and worn fasteners, components, settings, and material in scaffolding system	0.23167	37	0.001233	42

**Table 4:** Safety performance index calculation.

Category	Code	Name	Limiting vector	Limiting (normalized)	Evaluation of hazard	*L*_*n*_ *E*	SPI per category
			*L*	*L*_*n*_			
					*E*		
C1	C1H1	Insufficient working spaces/wrong site layout/no access/no lights	0.0123	0.0132	80	1.1	58.3
	C1H2	No housekeeping (scattered garbage and material, dusts, excessive noise, vibration, etc.)	0.0538	0.0579	50	2.9	
	C1H3	Insufficient food, drinking water, toilets, rest shelters, and medical facilities	0.0056	0.006	90	0.5	

C2	C2H1	Lack of company's safety policy	0.2799	0.3012	90	27.1	88.4
	C2H2	Insufficient safety training	0.0813	0.0875	90	7.9	
	C2H3	Insufficient safety motivation and incentives	0.0521	0.0561	80	4.5	
	C2H4	Negative management attitude to safety	0.035	0.0377	85	3.2	

C3	C3H1	Lack of proper planning and workforce for storage	0.0018	0.0019	70	0.1	78.7
	C3H2	Unsafe storage/stacking of materials and exceeding safe loading limits	0.0126	0.0136	80	1.1	

C4	C4H1	Failure in handling, inspection, and maintenance of equipment (cylinders, machines, hoses, and cables)	0.0016	0.0017	80	0.1	70.7
	C4H2	Lack of special PPE (personal protective equipment), such as face shield, special gloves, and goggles for welding/cutting	0	0	70	0	
	C4H3	Welders without training, license, and certificates	0.0036	0.0039	70	0.3	

C5	C5H1	Failure to perform form works under the supervision of a competent person	0.0053	0.0058	85	0.5	83.7
	C5H2	Use of weak and deformed forms	0	0	81	0	

C6	C6H1	Unavailability of a safe lift plan on-site	0.0024	0.0026	56	0.1	82.1
	C6H2	Lack of licensed trained riggers and operators	0.0056	0.006	55	0.3	
	C6H3	Lack of safe working load indicator/inspection stickers/latches/barricades	0.0218	0.0234	92	2.2	

C7	C7H1	Unsafe installation of the temporary power (old, damaged, and wrong rating of panels, sockets, wires, etc.)	0.0184	0.0198	81	1.6	71.7
	C7H2	Failure to apply access limit, lockout-tagout, permit system, and signage systems	0	0	61	0	
	C7H3	Lack of inspection for the electric tools, cables, and equipment	0.0073	0.0078	65	0.5	

C8	C8H1	Tools are in bad condition with no regular inspection.	0.0018	0.0019	73	0.1	90.4
	C8H2	Use of tools other than its intended use	0.003	0.0032	98	0.3	
	C8H3	Use or operation by untrained and unauthorized operators (lack of training system)	0.0149	0.016	91	1.5	
C9	C9H1	Failure to place adequate barriers and warning signs for open edges and holes	0.0194	0.0208	87	1.8	87
	C9H2	Falling of hand tools and other materials	0.0323	0.0348	84	2.9	
	C9H3	Failure to use required PPE (fall arrest systems) and safety nets	0.0512	0.0551	89	4.9	
	C9H4	Unsafe access to high places by damaged ladders, lifts, etc.	0	0	86	0	

C10	C10H1	Failure to provide appropriate and adequate personal protective equipment for workers (head, eye, face, hand, foot, and hearing protection)	0.0147	0.0158	96	1.5	96.8
	C10H2	Failure in enforcing, motivating, and training workers to use them	0.0733	0.0788	97	7.6	

C11	C11H1	Vehicles (buses/pickups/trucks/others) are in bad condition and do not have regular maintenance/first aid equipment/fire extinguishers/lights	0.0047	0.005	89	0.4	59
	C11H2	Failure in enforcing traffic regulations (seat belt, speed limit, license, and training)	0.0281	0.0302	54	1.6	

C12	C12H1	Use of defective and worn fasteners, components, settings, and material in scaffolding system	0.0012	0.0013	70	0.1	62.6
	C12H2	Lack of proper design, workmanship, and regular inspections	0	0	63	0	
	C12H3	Inadequate scaffolding stability (guardrails, toe boards, secured ties, etc.)	0.0096	0.0104	63	0.7	
	C12H4	Failure to provide safe access against slipping, sliding, or falling	0.0052	0.0056	60	0.3	

C13	C13H1	Lack of fire extinguishing training, escape plans, and drills	0.0028	0.003	80	0.2	72.2
	C13H2	Failure in controlling of ignition sources and fire watches, fire blankets, etc.	0.0054	0.0058	67	0.4	
	C13H3	Fire extinguishers are not enough, not in proper locations, not accessible, and not regularly maintained	0.0139	0.015	96	1.4	
	C13H4	Failure in storage of flammable liquids and combustible materials	0	0	60	0	

C14	C14H1	Wrong procedures (with slab, timber, trench, boxes, shoring, lining, etc.)	0.0034	0.0037	69	0.3	73
	C14H2	Failure to locate underground services and to take precautionary measures	0.0308	0.0332	70	2.3	
	C14H3	Lack of proper barriers/warning signs/lights for the excavation	0.0132	0.0143	81	1.2	
			0.929	1	3223	83.7	

## Data Availability

The data used to support the findings of this study are available from the corresponding author upon request.
